# Classifying sex with volume-matched brain MRI

**DOI:** 10.1016/j.ynirp.2023.100181

**Published:** 2023-07-20

**Authors:** Matthis Ebel, Martin Domin, Nicola Neumann, Carsten Oliver Schmidt, Martin Lotze, Mario Stanke

**Affiliations:** a University of Greifswald, Institute of Mathematics and Computer Science, Greifswald, 17489, Germany; b University Medicine Greifswald, Functional Imaging, Institute of Diagnostic Radiology and Neuroradiology, Greifswald, 17489, Germany; c University Medicine Greifswald, Institute for Community Medicine, Greifswald, 17475, Germany

**Keywords:** Sex discrimination, Machine learning, Convolutional neural network, Population based data, Voxel based morphometry

## Abstract

Sex differences in the size of specific brain structures have been extensively studied, but careful and reproducible statistical hypothesis testing to identify them produced overall small effect sizes and differences in brains of males and females. On the other hand, multivariate statistical or machine learning methods that analyze MR images of the whole brain have reported respectable accuracies for the task of distinguishing brains of males from brains of females. However, most existing studies lacked a careful control for brain volume differences between sexes and, if done, their accuracy often declined to 70% or below. This raises questions about the relevance of accuracies achieved without careful control of overall volume.

We examined how accurately sex can be classified from gray matter properties of the human brain when matching on overall brain volume. We tested, how robust machine learning classifiers are when predicting cross-cohort, i.e. when they are used on a different cohort than they were trained on. Furthermore, we studied how their accuracy depends on the size of the training set and attempted to identify brain regions relevant for successful classification. MRI data was used from two population-based data sets of 3298 mostly older adults from the Study of Health in Pomerania (SHIP) and 399 mostly younger adults from the Human Connectome Project (HCP), respectively. We benchmarked two multivariate methods, logistic regression and a 3D convolutional neural network.

We show that male and female brains of the same intracranial volume can be distinguished with >92% accuracy with logistic regression on a dataset of 1166 matched individuals. The same model also reached 85% accuracy on a different cohort without retraining. The accuracy for both methods increased with the training cohort size up to and beyond 3000 individuals, suggesting that classifiers trained on smaller cohorts likely have an accuracy disadvantage. We found no single outstanding brain region necessary for successful classification, but important features appear rather distributed across the brain.

## 1 Introduction

Brains of men and women are essentially similarly structured, showing only small differences in regional cortical and subcortical volumes, cortical thickness and white matter (Eliot et al., 2021) and belonging to the same population rather than two different ones (Joel and Fausto-Sterling, 2016; Joel, 2021). Nevertheless, large cohort studies (Lotze et al., 2019; Ritchie et al., 2018; Williams et al., 2021) and meta-analyses (Ruigrok et al., 2014; Sacher et al., 2013) confirmed that there are sex differences in global and regional brain volumes, which were, depending on study characteristics, partly corresponding and partly deviating from each other. Studies investigating sex disparities in other brain-related measures, such as cortical surface and thickness, structural and functional connectivity, and task-based fMRI, yielded mixed findings (for a review see Eliot et al., 2021). However, a recent large study based on data from the UK Biobank found sex differences in 67% of cerebral measures including brain volumes, thicknesses and surfaces, some of them also interacting with age (Williams et al., 2021). Even if effect sizes were small, this does not mean that these differences are meaningless, since small effects may be consequential in the long run (Funder and Ozer, 2019), and sex disparities in brain measures may be potentially powerful in interaction with other factors. Using multivariate statistical approaches, more subtle neuroanatomical sex differences can be uncovered (Sepehrband et al., 2018). Thereby methodological issues are crucial, such as the correction for overall brain size (Sanchis-Segura et al., 2020).

We here seek differences in the brains of males and females from the machine learning viewpoint (sometimes referred to as multivariate analysis) rather than from the statistical viewpoint. This means that we primarily seek accurate predictions rather than reliable and simply interpretable differences. The rationale is that machine learning methods are not bound by the rather strict assumptions of statistical models and can model complex relationships. If they are accurate and the problem of standardization or cross-dataset generalization can be solved, they can be used in primary care (Van Der Heijden et al., 2018). When additional methods to explain AI predictions (XAI) are mature and practicable, machine learning methods could further support care or research.

The addressed task is sex classification, i.e. to predict whether an MR image was taken from a man or woman. Previous studies reported that the brains of males and females can be differentiated with an accuracy of 69%–96% using voxel pattern analyses (Anderson et al., 2019; Brennan et al., 2021; Chekroud et al., 2016; Kurth et al., 2021; Feis et al., 2013; Sanchis-Segura et al., 2022). However, a large caveat to these accuracy numbers is that to our knowledge no large peer-reviewed study on sex classification followed the gold standard approach to correct for sex-specific average brain size differences by matching men and women of the same overall brain volume. The study of Wang et al. (2012) did so but had a sample of only 35 women and 35 men. Luders et al. (2009) used a volume-matched data set, but did not predict sex.

The average total intracranial volume (TIV) of men is larger than that of women, by about 12% according to Ruigrok et al. (2014). As is well-known and as we quantify below for our data sets, TIV therefore allows a fairly good discrimination between men and women. Sanchis-Segura et al. (2020) reported on their data set with a narrow age range that a sex prediction accuracy of remarkable 84% can be achieved using total intracranial volume (TIV) alone. To uncover subtle differences between brains of males and females, however, the total size difference is irrelevant. Thus, the overall brain size is a nuisance variable and should be corrected for to find relevant sex differences (Luders et al., 2009). Liu et al. (2014) argue that the popular normalization practice of dividing regional volumes by overall brain volume is not properly correcting for overall volume. They instead propose that a nonlinear correction term should be used and make a correction ansatz where the volume of interest is scaled by 1/TIV^*β*^, where *β* depends on the region and is estimated from the data (‘power-corrected proportions’). Many studies, such as ours, apply a non-linear spatial normalization of raw input in order to measure the volumes in a reference grid of voxels. This mapping is complex and it is not transparent how much information about the overall volume the different preprocessing procedures leave in the normalized data that could render the accuracies of different machine learning programs incomparable. Moreover, many studies do not describe such input normalization by overall brain volume, and therefore it must be assumed that it has not been accounted for. In this case, the reported accuracy values have very limited relevance to the question we pose. For example, the recent studies of Xin et al. (2019) and Luo et al. (2019) have reported accuracies of 93% and 96.7% but have not described a correction for overall brain volume. Eliot et al. (2021) summarize in their review that 8 out of 12 studies on sex prediction did not correct for brain size. Four further studies reported a decrease in accuracy to 59%–70% when brain size was controlled for with normalization (Chekroud et al., 2016; Sanchis-Segura et al., 2020, Sanchis-Segura et al., 2022; Zhang et al., 2018).

Several recent articles studied the influence of TIV-adjustment methods when searching sex-specific regional differences (Sanchis-Segura et al., 2019) or when predicting sex using multivariate methods (Sanchis-Segura et al., 2020, Sanchis-Segura et al., 2022; Wiersch et al., 2022). Sanchis-Segura et al. (2019) found that the TIV-adjustment method has a strong influence on the outcomes. Sanchis-Segura et al. (2020) found that, when no correction for TIV was performed, sex could be reliably predicted with >80% accuracy. However, after controlling for TIV variation with the power-corrected proportions method, the prediction accuracy dropped to about 60%. In a similar study, Sanchis-Segura et al. (2022) found that the best performing multivariate method they tried – random forests – classified 87.7% of images correctly when not correcting for TIV and 66% when they did with the power-corrected proportions method. In a recent preprint Wiersch et al. (2022) used principal component analysis and support vector machines to classify sex and report that the accuracy drops from 97% to 62% after removing confounding effects of TIV. Sanchis-Segura et al. (2020) even concluded that multivariate sex differences in gray matter volumes are largely dependent on male–female differences in TIV, a claim we dispute here using what Sanchis-Segura et al. (2019) referred to as the “only undisputed method to completely remove head-size variation”, a TIV-matched subsample. Remarkably, a recent preprint of Wiersch et al. (2022), which appeared three months after the preprint of this article, reported the same result we present here on a different dataset: Even after matching for TIV, sex can be predicted with at least 92% accuracy.

Even a highly accurate sex-classifying model or program is of limited relevance if it only performs well on the very same population of examples that it was trained on. Recently, Anaya-Isaza et al. (2023) employed convolutional neural networks (CNNs) to classify brain magnetic resonance images of the brain with respect to tumors and used transfer-learning to exploit parameters trained on one data set to learn parameters on a second data set. Here, we study the robustness of an MRI classifier, when the distribution of input data to be classified is different from the one the classifier was trained on, but no retraining is performed. Such generalizability is important for any method, targeting sex or a different predicted variable, that would eventually be applied clinically. In such a cross-cohort setting, classifiers need to deal with dissimilarities of used scanners, in silico data preprocessing methods, and population differences, in particular with respect to age and ancestry. Those factors can possibly cause a loss in prediction accuracy. Anderson et al. (2019) tested a weak form of generalizability using two cohorts. They studied a cohort of prisoners and a cohort of non-incarcerated people and obtained similar results on each. However, they trained in each case on the same data set they evaluated on and did not report any cross-cohort prediction accuracies. Regarding cross-cohort experiments, Eliot et al. (2021) found that “thus far, the only two [sex/gender] prediction studies to test their algorithms on external populations both found that their accuracy dropped to near chance levels”. Joel et al. (2018) measured cross-data set performance on test cohorts from three geographical locations different from the one of the training set. The cross-cohort accuracies were between 71% and 86%. More recently, Sanchis-Segura et al. (2020) performed a cross-cohort (external) validation, when they trained on a subset of narrow age range from the HCP and used the trained models to predict on their own data set with similar age distribution. Thereby, logistic regression and a simple artificial neural network achieved accuracies of 62% and 57% only. The development of robust methods that perform well cross-cohort has been challenging.

In this work, we demonstrate that sex can indeed be predicted with high accuracy from high-resolution T1-weighted MR imaging, even when the effect of the total brain size is completely removed by matching males and females by TIV. In addition, we break down the importance of individual regions of interest (ROIs) for our multivariate classifiers to test which areas of the brain contribute the most to the discriminatory power.

## 2 Methods

### 2.1 Characterization of data basis

In this study, we used two data sets from two different cohorts, the Human Connectome Project (HCP) and the Study of Health of Pomerania (SHIP). The SHIP data set includes data from the SHIP-2 and the SHIP-TREND-0 cohorts. Both cohorts include participants from the region of West Pomerania, Germany. SHIP-2 examinations were conducted from 2008 to 2012 and for SHIP-TREND-0 from 2008 to 2011 (Völzke et al., 2011). Both SHIP cohorts were pooled together, resulting in a data set of 3298 MRI scans. Participants' ages ranged from 21 to 90 years with a mean of 53 years. The mean age for female and male participants was 53 and 54, respectively. The age means do not differ significantly (*p* = 0.49, Mann–Whitney *U* test). The pooled SHIP data set has an almost balanced sex ratio (48.7% reported ‘male’ and 51.3% reported ‘female’). The overall mean TIV was 1505 ml (946–2209 ml), with 1609 ml for male and 1406 ml for female brains. The average TIV of men was 14.3% larger than that of women, matching approximately the aforementioned finding. The average GMV was 625.8 ml. The SHIP data set was primarily used for training and evaluation of the models.

**Fig. 1:**
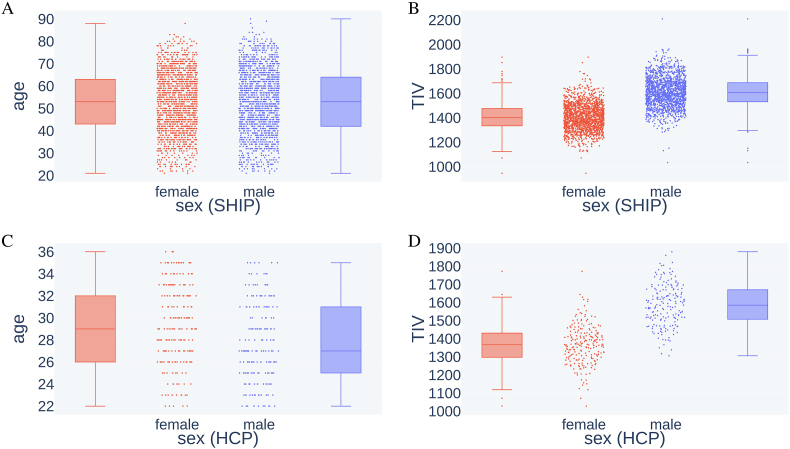
Data set properties age and TIV in male and female groups of both data sets. A) Age distribution in female and male samples in the SHIP data set. B) TIV distribution in female and male samples in the SHIP data set. C) Age distribution in female and male samples in the HCP data set. D) TIV distribution in female and male samples in the HCP data set.

To assess the cross-population performance of the models, we used the HCP S1200 data set of the Human Connectome Project (Van Essen et al., 2013). We removed twins and siblings from the original set to avoid biased prediction accuracies. The resulting HCP data set contained 399 T1w MRI scans, acquired from 2012 to 2015 on healthy young adults of age 22–36 (mean 29). The mean age of females was 29, of males 28. The age difference was significant in this data set (*p* = 8.82 ⋅ 10^−6^, Mann–Whitney *U* test). The sex was again near-balanced (with 46.4% reported males and 53.6% reported females). The overall mean TIV was 1468 ml (1028–1880 ml), with 1584 ml for male and 15.9% more than the 1367 ml for female brains. The average GMV was 719.1 ml. See Fig. 1 for an illustration of the properties of the data set.

The primary feature to be assessed by our proposed model was the voxel-wise gray matter volume (GMV) of high resolution T1-weighted anatomical MRI scans. For this purpose, tissue segmentation was performed using the SPM and CAT12 toolboxes running on the MATLAB platform. TIV was estimated using the output of the CAT12 toolbox. The following software versions were used: PM v7487, CAT 12.8.1 (v1987). At first, a spatial adaptive non-local means (SANLM) denoising filter (Manjón et al., 2010) improved signal-to-noise ratio, which was followed by internal resampling to properly accommodate low-resolution images and anisotropic spatial resolutions. The data were then bias-corrected and affine-registered to further improve the outcomes of the following steps, followed by the standard SPM “unified segmentation” (Ashburner and Friston, 2005). This acted as a starting point for the refinement stage. After skull-stripping, the brain was then parcellated into the left and right hemisphere, subcortical areas, and the cerebellum. Furthermore, local white matter hyperintensities were detected to be later accounted for during spatial normalization. Subsequently, a local intensity transformation was performed of all tissue classes, which is particularly helpful in reducing the effects of higher gray matter intensities in the motor cortex, basal ganglia, or occipital lobe before the final adaptive maximum a posteriori (AMAP) segmentation. This final AMAP segmentation step (Rajapakse et al., 1997), which is independent of *a priori* information of the tissue probabilities, was then refined by applying a partial volume estimation (Tohka et al., 2004), which effectively estimates the fractional content for each tissue type per voxel, encoded as a probability value between 0 and 1 for each tissue type. As a last default step, the gray matter (GM) tissue segments were spatially normalized to a common reference space (MNI152 NLIN, 2009c asymmetric) using Geodesic Shooting (Ashburner and Friston, 2011) registrations. As a spatial non-linear normalization removes most of the volume differences to the common template, the local volumetric changes due to this transformation have to be encoded. For this purpose, the Jacobian determinant of the local transformations was used to modulate each voxel intensity, which then encode dilation and contraction during that transformation to the common template space. As a result, after spatial normalization, the sum *∑*_*j*_ *x*_*j*_ of all voxel values, the *voxel sum*, of an individual equals his or her total gray matter volume up to small errors in the scale of 1 cm^3^. After spatial normalization, every voxel coordinate of each participant's GM tissue segment points to the same anatomical location, allowing a statistical comparison of location and GMV.

Afterwards, a reference template-based mask of GM tissue probability (thresholded to 0.5 and binarized) was applied to each single image to retain only highly probable GM voxels. Then, each single image was standardized with a Z-score normalization

(1) $${\tilde{x}}_{j}=\left(x_{j}-\mu \right)/\sigma ,$$

where *j* is the index of a voxel inside the masked brain and *μ* and *σ* are the mean and standard deviation of such voxel values in the image. For voxels *k* outside of the masked area, we set ${\tilde{x}}_{k}=0$. Training and classification was performed on the final preprocessed images $\tilde{x}$.

Z-score normalization was important not only as a way of feature scaling for the machine learning algorithms, it also removed the correlation of the sum of voxel values in each image with the TIV. Before Z-score normalization, the voxel sum and TIV showed a positive correlation with Pearson's correlation coefficient *r* = 0.706 (see Supplementary Fig. 1). Through Z-score normalization, the voxel values ${\tilde{x}}_{j}$ in each image sum up to zero, which removes this correlation. Furthermore, if the gray matter voxel values were proportional to GMV or TIV, then Equation (1) would ensure that all scans constitute identical inputs after normalization. However, Z-score normalization does not ensure that all information on the nuisance variable TIV is removed. In fact, Liu et al. (2014) argue that many brain structures' volumes are not proportional to, but obey a power law relationship with TIV. Therefore, it is possible that some partial information on TIV remains in the input even after normalization.

### 2.2 Machine learning classifiers

We compared two models for sex prediction, a convolutional neural network and logistic regression. Both models are parametrized functions that take as input an image of dimension *m* = 113 × 137 × 113 and output a *‘femaleness' score* *z* and a *‘femaleness’ probability* *p* = *σ*(*z*) ∈ [0, 1] (see Fig. 5), where *σ* is the logistic sigmoid function, i.e. *p* = 1/(1 + exp(−*z*)). We classified a brain as female if and only if *p* > 0.5 or equivalently if the femaleness score is positive (*z* > 0) and otherwise as male. The *accuracy* is computed as the percentage of samples, where the predicted and actual sex agree.

CNNs are routinely and successfully used in computer vision and image classification tasks. One benefit of CNNs over logistic regression is translation invariance, which means that features in input images can be recognized regardless of their positioning in the initial training images. If the data is distributed accordingly, they require fewer parameters and training images. On the other hand, for logistic regression to learn and recognize discriminative features in images, they must always be in the exact same position in the training set as well as in new inputs. However, logistic regression is less complex; thus it is quicker to train and the model parameters, one weight per voxel, are easier to interpret.

**Fig. 2:**
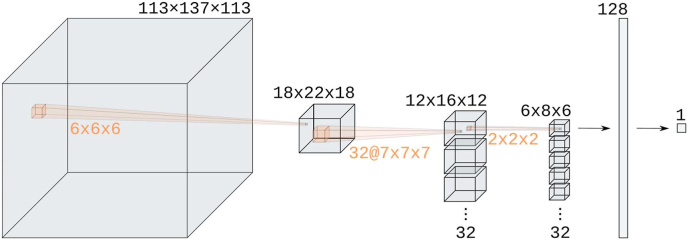
Illustration of BraiNN's architecture. The layer dimensions are written above the layers, and the pooling and filter dimensions are written in orange. The first layer is the input image, a *m* = 113 × 137 × 113 voxel MRI scan with one gray value per voxel. It is followed by a 6 × 6 × 6 max pooling (same stride), resulting in a 18 × 22 × 18 layer. To the result, a 7 × 7 × 7 convolution with 32 filters is applied, resulting in a 32 × 12 × 16 × 12 layer. This is again max-pooled with 2 × 2 × 2 (stride ‘same’), resulting in a 32 × 6 × 8 × 6 layer. This is flattened and fully connected to a 128 unit dense layer (left arrow). A dropout layer (not shown) with rate 0.5 is applied before the final dense layer (right arrow). The last layer outputs a single unit – the femaleness probability. (For interpretation of the references to color in this figure legend, the reader is referred to the Web version of this article.)

We call the CNN model BraiNN. BraiNN uses at its core the eponymous 3-dimensional convolutional layer. Additionally, it uses pooling layers, a dropout layer for regularization and two fully-connected (‘dense’) neural network layers as depicted and detailed in Fig. 2. The initial pooling layer was added to reduce the number of model parameters. We tried different pooling sizes and found that 6 × 6 × 6 pooling works well and with negligible impact on model performance (data not shown). The dropout rate in the dropout layer was 0.5. The activation function for the convolution and the 128 unit dense layer was PReLU (parametrized rectified linear unit). The second dense layer (the output layer) had a single unit with sigmoid activation function. In total, BraiNN has 1,264,769 parameters (or “weights”). BraiNN is open source and available from http://github.com/mabl3/BraiNN.

Logistic regression (here abbreviated as LogReg) uses one weight *w*_*i*_ for each input voxel value ${\tilde{x}}_{i}$ as well as a bias parameter *w*_0_. It computes the femaleness score very simply as

$$z=w_{0}+w_{1}{\tilde{x}}_{1}+\cdots +w_{m}{\tilde{x}}_{m}$$

and therefore requires to train 1,749,354 parameters. As training criterion, the cross entropy error was minimized. We used a L2-regularization term with the regularization parameter *λ* = 0.1, after asserting in a grid search that this value works well.

Note that we used – for computational convenience – a whole cuboid as input including the ∼76% of voxels that do not contain gray matter in any brain and that consequently do not vary between individuals. Note further that the final layer of BraiNN is equivalent to a logistic regression of the 128 features of the next-to-last layer that are a non-linear function of the image. Both models were implemented using the TensorFlow framework (Abadi et al., 2015). Training used a stochastic gradient descent algorithm (Adam optimizer, learning rate 0.0001).

The BraiNN and LogReg parameters were estimated on the SHIP data set during a 5 × 5-fold cross-validation as described in the next section. BraiNN was trained for 100 epochs on the complete cohort data set and for 200 epochs on the matched data set and in the ROI experiments, LogReg was trained for 30 epochs and for 60 epochs in the ROI experiments.

### 2.3 Cross validation

We used *k* × *ℓ*-fold cross-validation with *k* = *ℓ* = 5 for training, validation and evaluation. For this, the SHIP data set was split randomly and under the uniform distribution into five equally sized subsets. We then performed five “outer” training rounds, each time with a different one of the five subsets held back as a test set. The respectively remaining four subsets were combined to use for training. In each of the “outer” training rounds, we performed a nested cross validation by again randomly splitting the training data into five equally sized subsets. Again, five “inner” training rounds were performed, while holding back each time a different one of the five inner subsets as a *validation* set. The respectively four remaining inner subsets ((4/5)^2^ = 64% of the data) were combined to the actual training set. We then trained the model on the training set, using the validation set during training to monitor and control the training process. When the loss on the validation set did not decrease significantly (i.e. by more than 0.0001) for more than four epochs, the learning rate was reduced by multiplying it by 0.75. The model weights were stored each time the validation loss reached a new minimum. After the training was finished, the performance of the model was evaluated on the test set. For this, the weights from the point of the lowest validation loss during training were used. At each training round, it is ensured that the test and validation data were not used for parameter estimation and that the test data was never seen during the training procedure. This way, we trained the model 25 times on different random splits of the data set, and report the average performance of the single models on their respective test data as result. Performing cross-validation reduces random effects where a single split of the data into training, validation and test set works particularly well or bad and gives a more confident estimate of how well the model will perform on upcoming new data.

### 2.4 Human expert classification

We challenged an experienced MRI-researcher and neurologist (author Martin Lotze) to compete against LogReg in classifying the sex based on a subset of MRI scans. We randomly sampled 100 scans from the SHIP data set. He was allowed to look at the original complete MRI scans but sex information was withheld. We trained LogReg on the remaining 3198 SHIP images and let it classify the same 100 images as Martin Lotze. LogReg was only shown preprocessed images as before. The human expert principally had an advantage as the preprocessing for LogReg removed helpful information such as TIV, head shape and the size of the ventricular system.

### 2.5 Correction for TIV sex differences through matching

It is well known that brain size, and for that matter, body height, are correlated with sex (Ruigrok et al., 2014). In fact, the reported sex in our SHIP data set can be classified with 82.2% accuracy when simply classifying any scan below a TIV threshold of 1530.14 ml as female and otherwise as male. Accuracies of up to about 82% are therefore trivially achievable and irrelevant unless some correction for brain volume is performed.

**Fig. 3:**
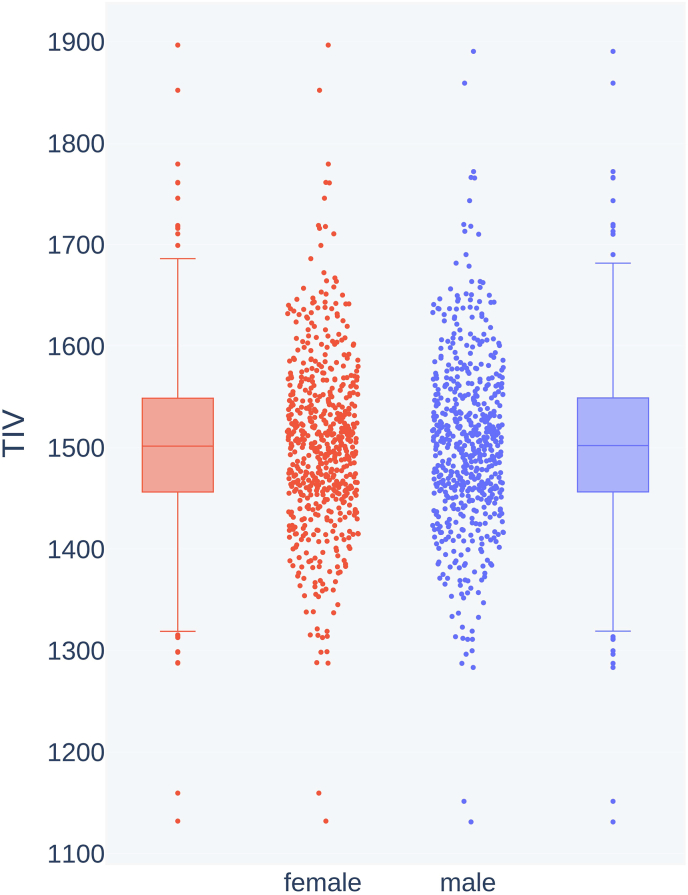
Distribution of TIV (vol, in cm^3^) in female and male MR images in the volume-matched data set.

Although the MRI scans are normalized for gray matter volume and should only encode local volume differences, we performed experiments on matched data sets to make sure the model does not benefit even from remains of global brain volume information. For this, the samples of the SHIP data set were grouped by sex and by TIV in steps of 10 ml. For each volume group, the largest of both sex subgroups was identified and a random sample of the same size as the smaller subgroup was taken. This way, we created a reduced ‘matched’ data set of 1166 images in which both sexes had virtually the same TIV distribution, shown in Fig. 3, and cannot be distinguished based on that parameter any more.

Since the matched data set is much smaller than the complete cohort data set (35%), a reduced performance of a parameter rich model is to be expected simply from the fact that fewer training images were available. To assess this effect, we also created a reduced data set by randomly sampling 1166 images from the complete SHIP data set and trained our models on this reduced data set. To reduce stochastic effects, we repeated the random sub-sampling 25 times. Each time, the training was performed with 5 × 5-fold cross validation as before. In this way, the models were trained 625 times on the reduced data set.

### 2.6 Regions of interest

We conducted experiments to investigate the contributions of certain brain regions to the accuracy of sex prediction. For this purpose and respecting prior neuroanatomical knowledge, we created 17 different binary regions of interest (ROI), compiled from 136 labels of the Neuromorphometrics atlas (provided by Neuromorphometrics, Inc. under academic subscription) as included in SPM12 and saved in the same dimensions as the MRI images. See Table 2 for the list of regions that are at least 1000 voxels large and supplementary file Neuromorphometrics_Derived_ROIs.xlsx for an exhaustive overview.

**Table 1:** Performance of LogReg and BraiNN in different training scenarios (‘Training Data’). In all cases, SHIP data was used for training. Consequently, HCP evaluations are cross-cohort. In the ‘Complete SHIP’ case, the entire SHIP data set was used for training (see Section 2). For ‘Matched SHIP’, the modified SHIP data set with equal TIV distributions for males and females was used (Section 2.5). In the ‘Reduced SHIP’ scenario, randomly sampled subsets of the entire SHIP data set were used with the same size as the ‘Matched SHIP’ data set. Accuracy denotes the fraction of correctly classified MRI scans at a femaleness score threshold of 0. AUC is the area under the receiver operating characteristic curve. Evaluations on SHIP were done using respectively held-back test data.

Training Data	Model	SHIP Accuracy	HCP Accuracy	SHIP AUC	HCP AUC
Complete SHIP	LogReg	95.78%	91.88%	0.994	0.983
	BraiNN	91.40%	85.17%	0.974	0.947
Matched SHIP	LogReg	92.71%	84.65%	0.978	0.940
	BraiNN	88.06%	76.37%	0.949	0.881
Reduced SHIP	LogReg	92.17%	87.69%	0.980	0.961
	BraiNN	87.32%	80.50%	0.948	0.918

**Table 2:** ROI based accuracy (AUC) of correct sex recognition, trained and tested on the volume-matched SHIP data set. Without any restricting to ROIs (‘whole brain’ at bottom) LogReg and BraiNN achieved AUCs of 0.978 and 0.949, respectively. When restricting LogReg for instance on the cerebellar ROI an AUC of about 0.95 was reached. The ROIs are sorted by decreasing AUC achieved by logistic regression.

	size	AUC when only 1 ROI is input	
ROI	in voxel	LogReg *↓*	BraiNN
Cerebellum	47928	0.95	0.89
Thalamus	5672	0.88	0.76
Frontal pole	13929	0.86	0.75
Amygdala Hippocampus	5816	0.84	0.76
Occipital lobe	28197	0.84	0.82
Basalganglia/Putamen	6891	0.82	0.68
Temporal gyri	32958	0.79	0.72
Medial frontal lobe	13244	0.79	0.71
Insula/posterior insula	5510	0.79	0.65
Paracentral gyri	23435	0.79	0.69
Medial parietal lobe	14112	0.78	0.74
Medial temporal lobe	1354	0.76	0.74
Frontal gyri	44235	0.75	0.73
Fusiform gyri	6343	0.75	0.66
Lateral parietal lobe	28126	0.75	0.72
Whole brain	426812	0.98	0.95

In one experimental setting (‘only ROI’), we applied each ROI mask so that only the respective brain region was left in the image, and the remaining voxels were set to zero. In a second setting (‘masked ROI’), we did the opposite, i.e. only setting the corresponding ROI voxels to zero and leaving the remaining image voxels unchanged. Thus, in the ‘only ROI’ setting, the classification results indicate how well the models perform on just small regions of the brain, and the ‘masked ROI’ classification results indicate how much the models *rely* on a brain region.

In the ‘only ROI’ results, a high AUC for one or more ROIs could indicate that the respective regions are sufficiently different between the sexes. In the ‘masked ROI’ results, a much worse performance compared to the models that used whole brain images would mean that the respective region was key to successful classification and thus could also indicate that the remaining regions have little information about the sex.

As logistic regression is an intrinsically interpretable, simple machine learning method, we pursued for LogReg yet another method to interpret which regions are important for the decision. The absolute value of a weight *w*_*j*_ itself indicates whether voxel *j* is an important contributor to the models decision and the sign of the weight indicates whether more gray matter at *j* makes a woman or man more likely. Let *σ*_*j*_ be the standard deviation of ${\tilde{x}}_{j}$ over all samples. We normalize the voxel weights via $w_{j}^{\prime }=\sigma_{j}w_{j}$ to obtain the weights relative to the locally observed variation and obtain the same predictions as if we had standardized the variance of voxel values in the first place:

$$z=w_{0}+\underset{j}{\sum }w_{j}{\tilde{x}}_{j}=\underset{j}{\sum }w_{j}^{\prime }\frac{{\tilde{x}}_{j}}{\sigma_{j}}.$$

In the 5 × 5 cross validation we obtain 25 sets of weights. For visualization purposes, the histograms of the 25 LogReg models were split into two maps (negative values multiplied by −1) and summed up voxelwise, respectively. Then, both maps were smoothed using a Gaussian smoothing kernel (1 mm FWHM), interpolated into a 0.5 × 0.5 × 0.5 mm^3^ space, scaled to 1 by dividing by the maximum value and the final maps multiplied by 25.

## 3 Results

### 3.1 Performance using complete cohorts

First we compared the two machine learning classifiers BraiNN and LogReg with each other using all – and therefore unmatched – data of the complete SHIP cohort as described in Section 2.3. Recall that the normalization described in Section 2.1 is already supposed to partially control for overall brain size. The average performances are shown in Table 1. LogReg performed better on the SHIP test data with 95.78% accuracy, but BraiNN also got a high accuracy of 91.40%. This is also mirrored in the area under the receiver operating characteristic curve (AUC). LogReg had an AUC of 0.994, and BraiNN of 0.974 on the SHIP test data. The left of Supplementary Fig. 2 shows the ROC curves.

The LogReg accuracy is just below the maximum reported accuracy we found in the literature: Feis et al. (2013) had reported a sex classification accuracy of 96%, achieved with a support vector machine. However, their study did not describe any control for overall brain size, indicating that this comparison may not be fair. Additionally, their data set contained only *n* = 67 + 55 individuals, suggesting a relatively low precision of the accuracy estimate.

### 3.2 On volume-matched participants

**Fig. 4:**
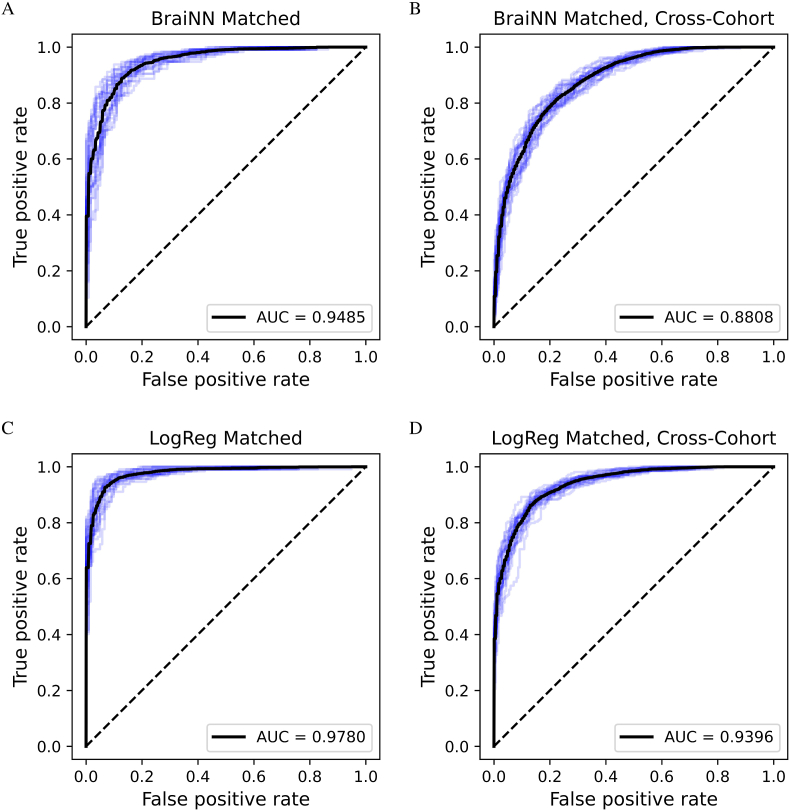
Receiver operating characteristic (ROC) curves for BraiNN and LogReg on the matched SHIP data set. The ROC curves of each single training run are shown in blue, the black curves are the respective averaged ROC curves. The mean area under the curve (AUC) is shown in the bottom right of the plots. A) ROC and AUC for BraiNN, trained on the matched SHIP data set when predicting the SHIP test data. B) ROC and AUC for BraiNN, trained on the matched SHIP data set when predicting the HCP data set. C) ROC and AUC for LogReg, trained on the matched SHIP data set when predicting the SHIP test data. D) ROC and AUC for LogReg, trained on the matched SHIP data set when predicting the HCP data set. (For interpretation of the references to color in this figure legend, the reader is referred to the Web version of this article.)

**Fig. 5:**
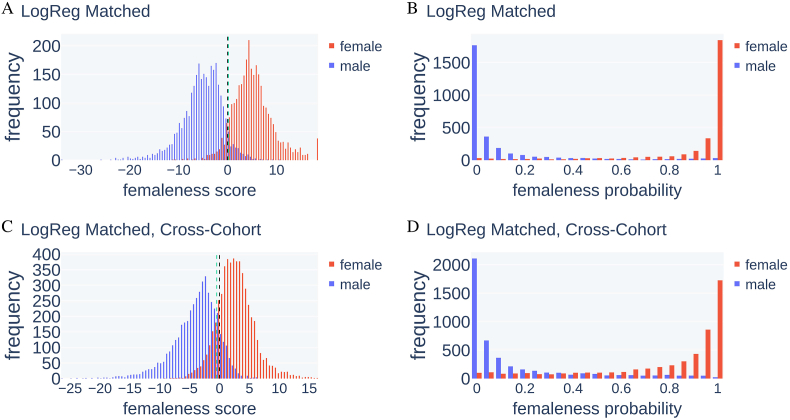
Distribution of femaleness score and femaleness probability from LogReg on SHIP test images and the HCP data set after training on the matched SHIP data set. A) Distribution of the femaleness score, red bars depict the frequency (y axis) of the score (x axis) for female scans, blue for male scans. A score below 0 (black dashed line) leads to classification of the image as male, otherwise as female. B) Distribution of the femaleness probability. Scans with a femaleness probability above 0.5 are classified as female. C) Distribution of the femaleness score when predicting on the HCP data set. D) Distribution of the femaleness probability when predicting on the HCP data set. The green dashed line is drawn at the decision threshold that maximizes classification accuracy. The optimal thresholds are near a score of 0 which is equivalent to a threshold of 50% femaleness probability. (For interpretation of the references to color in this figure legend, the reader is referred to the Web version of this article.)

In order to ensure that possible undetected remains of global brain volume information in the data do not distort the models, we then used the matched SHIP data set described in Section 2.5 for training LogReg and BraiNN. Both methods were evaluated using 5 × 5-fold cross-validation. As can be seen from the results in Table 1, LogReg still performed better on the SHIP test data with an accuracy of 92.71% compared to BraiNN with 88.06%. The AUCs for the matched data set showed the same trend as with the complete cohort data set. LogReg had an AUC of 0.978, and BraiNN of 0.949. See the left side of Fig. 4 for the ROC curves. The distribution of the femaleness score *z* and the femaleness probability *p* output by LogReg for the test images are shown in Fig. 5 A and B. The femaleness score distributions peaked at around −5 and +5 for male and female images, respectively. Both distributions appeared to approximately follow a normal distribution. LogReg correctly and confidently predicted the sex of most test images. There were very few extreme cases of women classified as very likely male and vice versa. However, the shape of Fig. 5A does not suggest that there is a substantial fraction of label errors, in which case either distribution would be expected to have a second mode. The femaleness probability was correctly close to 0 or 1 for most images and the default classifier threshold of 0.5 is near-optimal in this setting. Supplementary Fig. 9 shows the distribution of femaleness scores and probabilities predicted by BraiNN. They were generally similar but a little less clearly separated between sexes.

Panels C and D of Fig. 5 show the femaleness score and probability for LogReg when classifying the HCP images cross-cohort. The femaleness scores for women and men have a higher overlap, and the distributions were shifted and now peak around −4 and +4 for male and female images, respectively. Here also most brains from women had a femaleness probability close to 1 and brains from men close to 0.

**Fig. 6:**
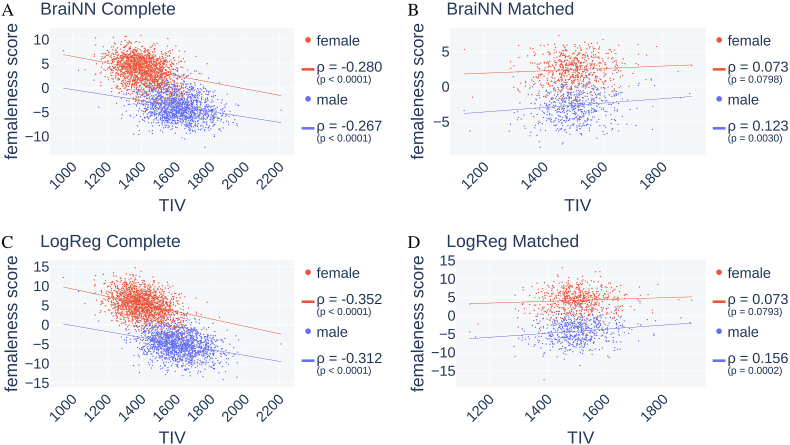
Femaleness score vs. TIV of test images from BraiNN (A, B) and LogReg (C, D) on SHIP test images after training on the complete (A, C) or matched (B, D) SHIP data set. Red and blue dots represent individual MR images from women and men, respectively. In red and blue are the regression lines for the female and male samples, respectively. Pearson's correlation coefficients *ρ* for femaleness and TIV within sex and cohort are given in the legends, as well as the corresponding p-values from a two-sided Wald test. (For interpretation of the references to color in this figure legend, the reader is referred to the Web version of this article.)

Fig. 6 and Supplementary Fig. 3 visualize the influence of TIV on prediction accuracies on within-cohort and cross-cohort training, respectively. In the two plots on the left, the models were evaluated (cross-validated) on the complete data set after being trained on the complete SHIP data set. For all eight combinations of sex, ML method and test data set, and without size-matching, the models have a tendency to classify larger brains as rather male, i.e. with a lower femaleness score. Within cohort, the effect is a bit stronger in LogReg with a Pearson's correlation coefficient *ρ* of −0.352 (*p* = 1.2 ⋅ 10^−50^, two-sided Wald test) and −0.312 (*p* = 1.3 ⋅ 10^−37^) for females and males, respectively, and −0.280 (*p* = 8.2 ⋅ 10^−32^) and −0.267 (*p* = 1.2 ⋅ 10^−27^) for BraiNN. As is apparent from the plots on the left of Fig. 6, when trained on the unmatched data and if the sex of an individual is given, either model can be used to predict TIV via the individual's femaleness score using their correlation. Consequently, TIV information is somehow contained at least partially in the data even after standardization. This could be due to a nonlinear relationship between total and local volumes (Liu et al., 2014). Regardless of the concrete explanation, this gives evidence that the standardization is not a sufficient provision against unwanted exploitation of TIV information for sex classification. This supports our overall argument for using a TIV-matched data set instead. When the models were trained on the TIV-matched data set (right-hand plots), there was a reversed effect in all eight combinations of sex, method and cohort: a slight and sometimes even insignificant tendency to classify larger brains as more female. For LogReg, the respective correlation coefficients for female and male were 0.073 (*p* = 0.0793) and 0.156 (*p* = 0.0002), while for BraiNN they were 0.073 (*p* = 0.0798) and 0.123 (*p* = 0.0030). I.e., larger brains, regardless whether from a woman or man, have a slight tendency to get a higher femaleness score, when trained on TIV-matched brains. Note that in the matched setting, TIV is by design uninformative to predict sex and therefore partial information about TIV cannot be exploited to directly predict the sex.

We also examined the influence of age on the classification. Supplementary Fig. 4 C shows that there is a slight tendency that older individuals are predicted as rather male; with LogReg *ρ* = −0.18 (*p* = 1.3 ⋅ 10^−5^) for women and *ρ* = −0.19 (*p* = 3.7 ⋅ 10^−6^) for men. However, the classification accuracy is high over the full range of decades of life 20–29, …, 80–89.

Previously, Wang et al. (2012) had performed sex classification on a volume-matched data set of only 35 male and 35 female subjects. Their support vector machine had as input combined structural MRI and resting-state fMRI data and obtained a mean classification accuracy of 89%. Our somewhat higher accuracy confirms the result that even with a careful gold standard control for brain volume the sexes can be distinguished quite accurately. Furthermore, our much larger data set of 1166 images allows for more precise estimates of the accuracy and we here used only part of the input features that Wang et al. (2012) had used.

### 3.3 Influence of training cohort size

Using a volume-matched subset in Section 3.2 reduced the prediction accuracy of both studied methods with respect to using the complete SHIP cohort in Section 3.1. As hypothesized, this could be a consequence of what is sometimes called *feature leakage* in the machine learning domain. A feature not intended to be used for prediction, here TIV, contains, as argued above and even after normalization, at least partial information on the value we want to predict. On the other hand, smaller training sets also generally lead to less accurate models, when their number of parameters is large. To assess the impact of the latter, i.e. of the reduced number of training images, we also trained the models on randomly sampled subsets from the SHIP data set. As one of the subset sizes we chose the size of the matched data set, 1166, in order to make comparisons that allow to disentangle the two expected causes for said difference.

**Fig. 7:**
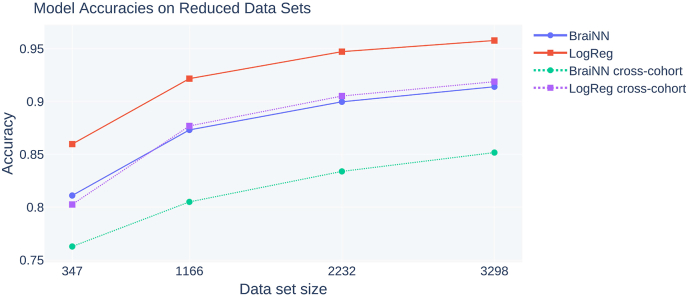
Influence of the data set size (347, 1166, 2232 and 3298) on the model's AUC. The smaller sized data sets were randomly sampled from the complete (unmatched) cohort data set (3298) and training was performed as described in Section 2.5.

Fig. 7 shows the accuracies of our methods as a function of cohort size. Note that with the cross-validation we use, a larger cohort size both allows a better parameter training and a more precise evaluation of the accuracy.

In comparison, the performance on the randomly reduced data set of the same size of the matched data set was 92.17% (matched: 92.71%) accuracy for LogReg and 87.32% (matched: 88.06%) for BraiNN on the SHIP test data. Thus, matching for TIV resulted for both methods in a similar loss of accuracy as can merely be expected from the resulting reduction of the training set size. Apparently, the partial information about TIV that remains after normalization could not be exploited by either method for a significantly improved accuracy. However, this may be different for other methods. Naturally, if not done with matching, the choice of a brain-size normalization procedure may have a large influence on sex predictability (Sanchis-Segura et al., 2020).

The leftmost data points in Fig. 7 show the accuracy for a data set size of 347, which is the number of children of which Kurth et al. (2021) classified their sex using relevance vector regression (RVR) following a principal component analysis (PCA). On their data set Kurth et al. achieved a classification accuracy of 80.4%. When we downsample our data set to the same size in order to correct for advantages from our larger data set, LogReg and BraiNN achieve accuracies of 85.97% and 81.11%, respectively. This suggests that our machine learning approaches may be competitive with or better than the approach of Kurth et al. (2021).

### 3.4 Cross cohort prediction

A major concern for translation of machine learning (ML) methods to clinical use is their robustness with respect to differences in machines, protocols, preprocessing software and practices. An ML method could perform better than a human expert when applied to data from the same distribution as the data it was trained on but perform poorly and much worse than an expert when applied to data, say, from a different machine. In order to study the robustness of our methods we used a second data set with data from the Human Connectome Project (HCP), which is unrelated to the SHIP cohort and its age distribution is narrower and with a much smaller mean. This allows to assess how well the trained models generalize to new images from a different scanner and population. We therefore performed between-data set experiments. SHIP images were used for training and HCP data for performance evaluation. Again, 5 × 5-fold cross-validation was performed. Table 1 shows the results for logistic regression and our CNN.

As expected, in a cross-cohort prediction the accuracy dropped for both methods compared to the within-cohort results (Table 1). When training on the complete SHIP cohort and predicting on HCP, the LogReg performance dropped to 91.88% accuracy. The AUC also dropped, to 0.983 (ROC on the right of Supplementary Fig. 2). BraiNN retained an accuracy of 85.17% (AUC 0.947). When trained on the volume-matched SHIP data set, the accuracies on the HCP cohort were 84.65% for LogReg and 76.37% for BraiNN (AUC 0.940 and 0.881, respectively, see right side of Fig. 4).

### 3.5 Regions of interest

We performed the ROI experiments on both the complete cohort and matched SHIP data set. We observed overall the same effects in both cases and will therefore only describe the matched data set results in more detail. The other results can be found in the Supplementary Material.

The first experimental setting ‘only ROI’, where only one region is ‘visible’ to the classifier, revealed that for both models any of the investigated single brain regions larger than 1000 voxels were enough to classify brains with an AUC of at least 0.65 (see Table 2). While slightly better than random guessing, this was a poor performance compared to the whole brain images, where LogReg and BraiNN achieve 0.978 and 0.949, respectively. However, some ROIs performed quite well on their own. Especially the cerebellum showed the highest AUC for both models with 0.953 and 0.886 for LogReg and BraiNN, respectively. When classifying the HCP data set, the cerebellum also performed best with an AUC of 0.894 and 0.812 for LogReg and BraiNN (whole brain images: 0.940 (LogReg) and 0.881 (BraiNN)).

**Fig. 8:**
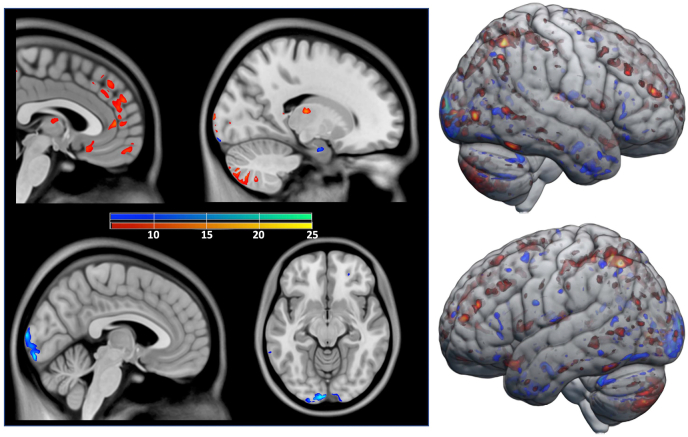
Regions that are important for sex discrimination with logistic regression on the matched data set. An increase in gray matter volume of voxels in red or yellow makes ‘woman’ more likely, an increase in gray matter volume of voxels in blue or green makes ‘man’ more likely. The color gradients red-to-yellow for women and blue-to-green for men give the repeatability among 25 training repetitions and are thresholded at 7. Cortical and subcortical regions have been overlayed on slices (left part); brain surface regions have been overlayed on a rendered brain (right part). For detection of a female brain thalamus, medial prefrontal lobe (mPFC), orbitofrontal cortex (OFC), inferior cerebellar hemisphere and the intraparietal sulcus showed highest importance. For the detection of a male brain amygdala, occipital pole and inferior temporal lobe showed highest importance. (For interpretation of the references to color in this figure legend, the reader is referred to the Web version of this article.)

As depicted in Fig. 8 most overlaps of logistic regression procedures were observed for the recognition of femaleness (red-orange) in the thalamus, medial prefrontal lobe (mPFC), orbitofrontal cortex (OFC), inferior cerebellar hemisphere and the intraparietal sulcus. For increased maleness (blue-green), amygdala, occipital pole and inferior temporal lobe showed highest overlap.

Supplementary Fig. 5 shows a bar plot of the model performances on the SHIP and HCP data sets. The exact values are shown in Supplementary Table 3. Other regions with an AUC >0.8 (LogReg, BraiNN) were occipital lobe (0.837, 0.821), frontal pole (0.857, 0.746), thalamus (0.881, 0.764), amygdala hippocampus (0.845, 0.762) and basalganglia (0.815, 0.677).

The second experimental setting (‘masked ROI’) showed for both models and both training data sets only slight differences in model AUC, i.e. leaving out any ROI could be compensated by the models and performed almost as good as when the whole brain was input. See the Supplementary Material for bar plots illustrating the different AUCs for “whole brain” images and images with isolated or masked ROIs for both models and both training data sets, and the performances on the cross cohort data (Supplementary Figs. 6–8). The exact values are shown in Supplementary Tables 2, 4 and 5.

### 3.6 Machine versus human expert

Of the 100 test scans given both to LogReg and Martin Lotze, LogReg correctly classified 94 scans and ML correctly classified 62 scans. 35 images were correctly classified by the program and not by the expert, whereas 3 images were correctly classified by the human and not by the program, 59 were correctly predicted by both. LogReg is more accurate at sex classification than this expert (*p* < 7 ⋅ 10^−8^, two-sided sign test).

## 4 Discussion

We examined the question whether two same-size brains, one of a male and one of a female, are distinguishable from magnetic resonance images and how the sex classification accuracy of a machine learning model depends on the method, the cohort size and whether the method's parameters were estimated on the same or a different cohort. The extent to which the brains of men and women differ had been studied extensively and discussed controversially. Recently, it has been argued that the human brain is not sexually dimorphic (Eliot et al., 2021), that the sex differences can largely be attributed to male-female differences in TIV and that TIV-adjustment methods can be ineffective and influence much the results (Sanchis-Segura et al., 2020). Analyses in the MRI domain of machine learning applications are also impeded by the fact that one team of researchers does (can) not reproduce the results of another team of researchers. Comparisons become inconclusive, e.g. when another cohort is used, other scanners, other TIV-adjustment methods as well as other classification methods and if the respective program's code, version and calling parameters are not available.

We found that indeed same-size brains of men and women can be distinguished with high accuracy. At least an accuracy of 92% can be achieved on a data set where males and females of the same TIV were matched. Thereby, the differences between the sexes cannot be explained away by differences in overall volume. In our large data set of 1166 volume-matched participants this accuracy also cannot be explained by chance. Therefore, the information to distinguish the brains of men and women is contained somehow in their MR images. Our methods achieve a better accuracy than a human expert but have the disadvantage that they do not intrinsically explain their decision for an individual. Our high accuracy when matching TIV is in contrast to the results of (Sanchis-Segura et al., 2022), who analyzed to what extent a multivariate prediction method's performance is affected by TIV-adjustment and found that it dropped to 66%.

Our convolutional neural network performed worse than logistic regression despite the fact that CNNs are designed for computer vision tasks and are widely and very successfully used for deep learning on images. We believe this may be because their advantage of being able to efficiently learn translation-invariant patterns is not important for our MR images. Rather, the spatial registration procedure that is possible and customary with brain MRIs gives individual voxels a meaning that is comparable between images. Logistic regression on individual voxel values exploits this property but only with a simple linear function. More general non-linear model classes, e.g. multilayer perceptrons, could perform even better, what may be an objective for further research.

The TIV of the test images and their respective femaleness score correlated within each sex with *ρ* ≈ − 0.3 when cross-validated on the *unmatched* data set (Fig. 6C). This suggests that information on TIV was not completely removed by the normalization and may be exploited on the unmatched dataset by the model to classify small brains rather as female. In contrast, the information on TIV, which is left after normalization, could not be used to predict sex in the matched dataset. This strengthens the notion that the models indeed learn meaningful discriminatory features and are less dependent on features that are correlated with overall brain size.

We tried to order the relative importance of different regions of the brain for sex discrimination with logistic regression by occluding everything but a given ROI as input during prediction and also during *training*. We found that cerebellum, thalamus, frontal pole, amygdala/hippocampus and occipital lobe are contributing most to the discriminatory power. This ROI-approach is fitting well to the voxel-based approach as depicted in Fig. 8. Here, for detection of a female brain the thalamus, medial prefrontal lobe (mPFC), orbitofrontal cortex (OFC), inferior cerebellar hemisphere and intraparietal sulcus showed highest importance. For the detection of a male brain the amygdala, occipital pole and inferior temporal lobe were most important. Interestingly, cerebellum, temporal gyri and occipital lobe have been the areas exhibiting larger GMV in males than in females in a previous paper (Lotze et al., 2019) and have been connected to sensorimotor (cerebellar hemispheres), (higher) visual recognition and perception (fusiform gyrus and occipital lobe) but also to cognitive processing (STS, temporal pole).

The differences between brains of females and males are, however, likely not restricted to size differences of certain regions of interest but more complex. None of the regions are by themselves necessary for discrimination and nearly the same accuracy can be achieved when each region is occluded versus when the whole brain is visible. We believe that more advanced methods for *explainable* artificial intelligence are required to assist an expert in interpreting sex differences beyond mere regional size differences, to explain why a particular brain belongs to the predicted class and to fill the gap of unexplained differences that statistical methods of per-region GMV leave. Such explanations of the decisions of an accurate classifier could help to shed light on diseases with a large gender effect, e.g. ones related to pain. Sex can serve as an easily available example trait to benchmark methods with larger training cohorts of ‘affected’ individuals than for many disease traits. Studies of predicting disease traits would have to address the same questions and issues like the effect of cohort size on accuracy and generalizability to other cohorts.

We show how the classification accuracy increases with the size of the data set available for training. In particular for smaller cohorts, say of a few hundreds participants or below, the accuracy decreases significantly. This means that some of the true sex differences may not be uncoverable in studies on smaller cohorts. This also means that comparisons between methods should take different data set sizes into account. As one can draw inferences from a normalized image (via the femaleness score) about TIV we conclude that indeed – and despite our best effort – our complete cohort data set appeared to suffer from feature leakage, where our preprocessing with spatial and Z-Score normalization did not succeed to fully remove the information from the nuisance variable TIV.

An ultimate goal may be that a machine learning method can produce valuable information from an MRI scan, without requiring that thousands of other individuals from the same population have been scanned before on the same machine. Toward that goal we studied the loss in accuracy that is obtained when training on one cohort and evaluating on another cohort. The drop in accuracy for cross-cohort prediction may be explained by the fact that – strictly speaking – sex prediction on another cohort is another ML task that the predictor was not trained for. For example, it is possible that scanner differences were a main cause of the reduced accuracy. The cross cohort results are likely sensitive to distributional differences between the cohorts and the generalizability would ideally be tested using scans from many cohorts or other heterogeneous sources.

## 5 Limitations of the study

The ROIs we chose for the occlusion experiments are rather coarse. Further examinations could possibly improve interpretability by using a finer partition of the brain or a ‘searchlight’ approach (Weaverdyck et al., 2020).

The sex attribute was taken from the locals residents registration office. It is assigned after an external examination of body characteristics at birth and may not always reflect gender identity. We carefully excluded the information that brain size holds on sex. Also, male and female individuals had very similar age distributions in the SHIP cohort. Furthermore, all ROIs allow some discrimination by themselves and the sex information is encoded broadly over the brain. This is an argument that neuroanatomy in the narrower sense is indeed the factor modulating correct classification, as opposed to, say, sex-specific training effects of the brain. However, we cannot exclude the possibility that some other attribute of individuals – unknown to us or unrecognized – correlates with sex and is predictable from MR images. Such an attribute could be irrelevant to the question whether and how male and female brains differ *principally*, e.g. because it's influenced by a person's behaviour. For example, in the SHIP cohort it has been shown that sex is correlated with cardiorespiratory fitness which itself is correlated within a sex with regional gray matter volumes and total brain volume (Wittfeld et al., 2020). A classifier may use such a feature successfully even though it may depend on the population and it may not be universally discriminatory between sexes.

## 6 Conclusions

The current study set out to investigate if sex can be predicted from gray matter volumes when total brain size is completely removed by matching males and females by total intracranial volume. In a large data set, we were able to achieve high classification accuracies of 92% on the training cohort and of 85% when evaluated on another cohort. Cross-validation results on the complete cohort of ∼3300 individuals even achieved an accuracy of >95%. Multivariate approaches are indeed able to discriminate between brains of males and females.

## 7 Contributions

me and ms planned the study, performed the analyses and wrote the manuscript. md and ml planned the study and wrote the manuscript. nn performed the literature search and wrote the manuscript. cos managed the data.

## Declaration of competing interest

The authors declare that they have no known competing financial interests or personal relationships that could have appeared to influence the work reported in this paper.

## Data Availability

The data that has been used is confidential.
