# Supplementary material for: Classifying sex with volume-matched brain MRI
Source: Neuroimage Rep. 2023 Jul 20;3(3):100181. doi: 10.1016/j.ynirp.2023.100181 (PMC12172721; doi:10.1016/j.ynirp.2023.100181)
Supplement: Multimedia component 1 [file mmc1.pdf]

Classifying sex with brain MRI
Supplementary Material

Matthis Ebel[1], Martin Domin[2], Nicola Neumann[2], Carsten Oliver Schmidt[3], Martin Lotze[2], and Mario Stanke[1]

[1]Institute for Mathematics and Computer Science, University of Greifswald, Walther-Rathenau-Str. 47, 17489, Greifswald, Germany
[2]Institute of Diagnostic Radiology and Neuroradiology, Functional Imaging, University Medicine Greifswald, 17489, Greifswald, Germany
[3]Institute for Community Medicine, University Medicine Greifswald, 17475, Greifswald, Germany

June 19, 2023

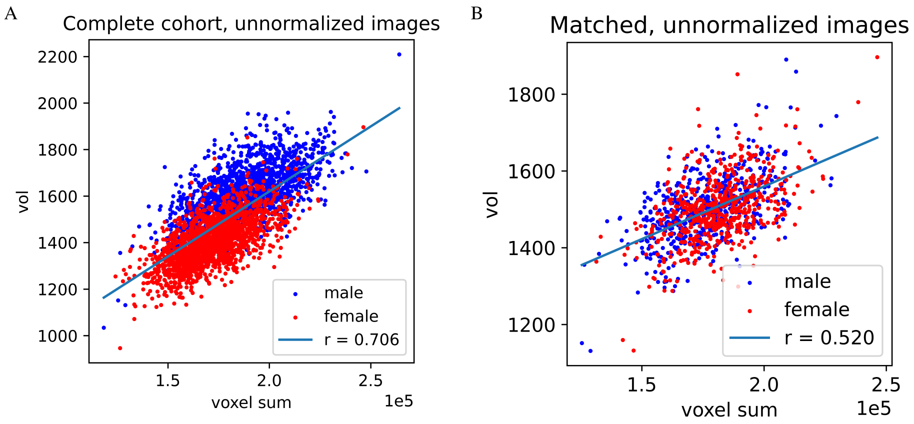

Supplementary Figure 1: Correlation of voxel sum in each MR image with the TIV (vol). Blue dots represent male samples, red dots female. The straight line shows a linear regression calculated on all images (male and female pooled), 'r' is Pearson's correlation coefficient. A) Correlation for the complete SHIP data set for images that have not been Z-score normalized. B) Correlation for the TIV-matched data set for images that have not been Z-score normalized.

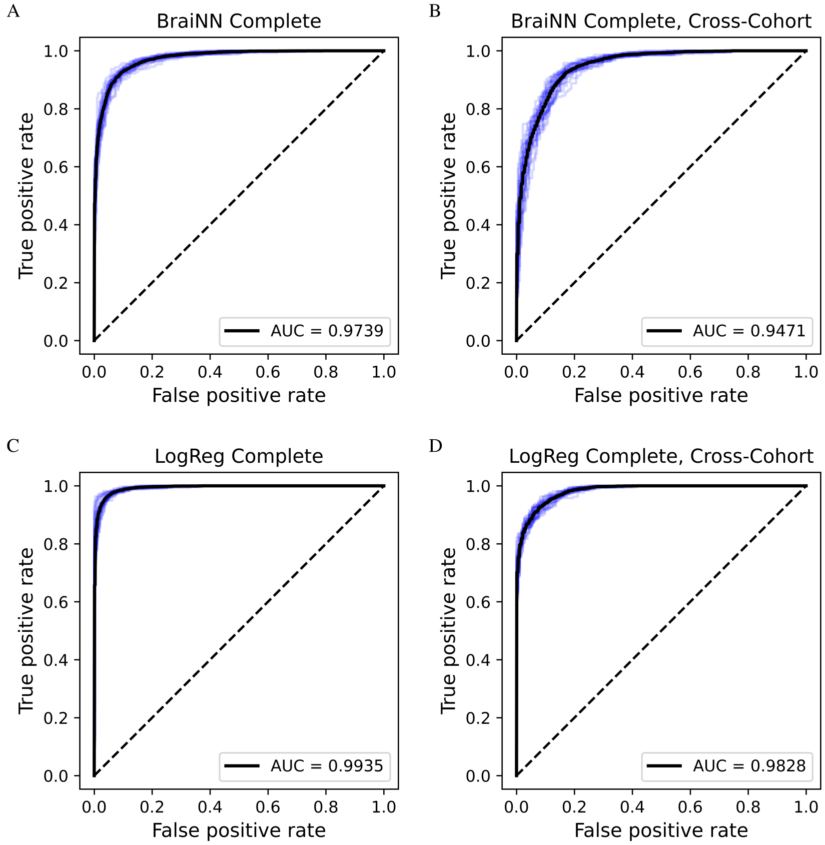

Supplementary Figure 2: Receiver operation characteristic (ROC) curves for BraiNN (CNN) and LogReg on the complete SHIP data set. The ROC curves of each single training run are shown in blue, the black curves are the respective averaged ROC curves. The mean area under the curve (AUC) is shown in the bottom right of the plots. A) ROC and AUC for BraiNN, trained on the complete SHIP data set when predicting the SHIP test data. B) ROC and AUC for BraiNN, trained on the complete SHIP data set when predicting the HCP data set. C) ROC and AUC for LogReg, trained on the complete SHIP data set when predicting the SHIP test data. D) ROC and AUC for LogReg, trained on the complete SHIP data set when predicting the HCP data set.

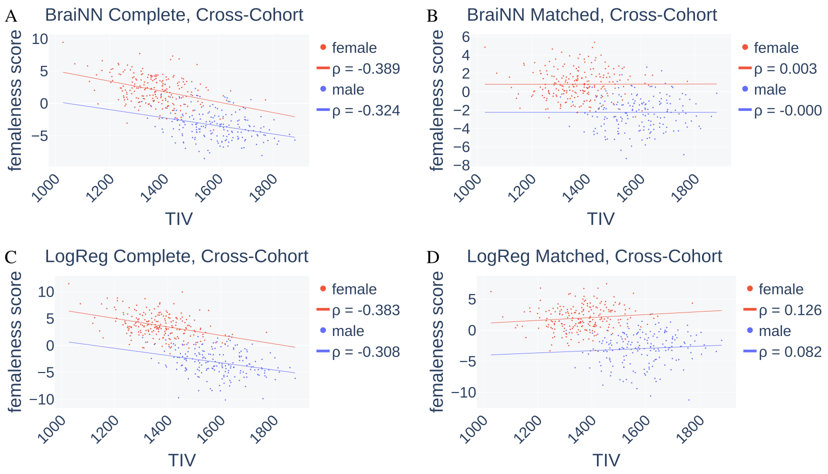

Supplementary Figure 3: Femaleness score vs. TIV of test images from BraiNN (A, B) and LogReg (C, D) on HCP test images after training on the complete (A, C) or matched (B, D) SHIP data set. Red and blue dots represent individual MR images from women and men, respectively. In red and blue are the regression lines through the female and male samples, respectively. Pearson's correlation coefficient ρ given in the legends.

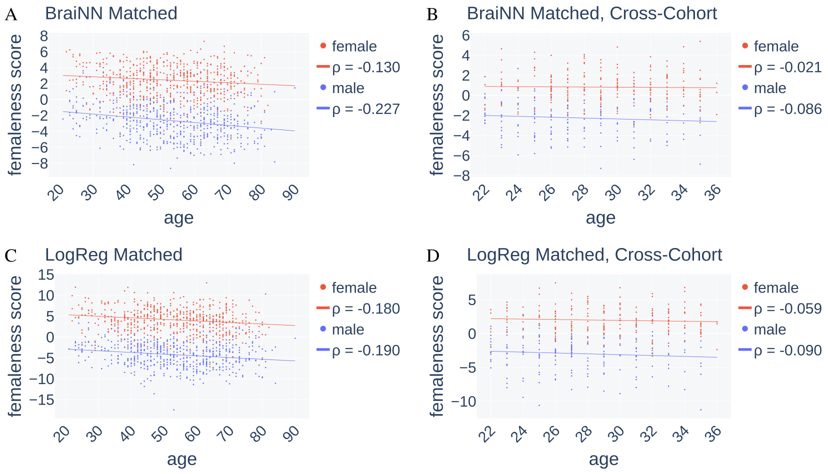

Supplementary Figure 4: Femaleness score vs. age of test images from BraiNN (A, B) and LogReg (C, D) on SHIP (A, C) and HCP cross-cohort (B, D) test images after training on the matched SHIP data set. Red and blue dots represent individual MR images from women and men, respectively. In red and blue are the regression lines through the female and male samples, respectively. Pearson's correlation coefficient ρ given in the legends. The respective p-values for the correlation in female and male are A) 0.0017 and $2.81 \cdot 10^{-8}$, B) 0.7648 and 0.2419, C) $1.29 \cdot 10^{-5}$ and $3.68 \cdot 10^{-6}$, D) 0.3896 and 0.2216.

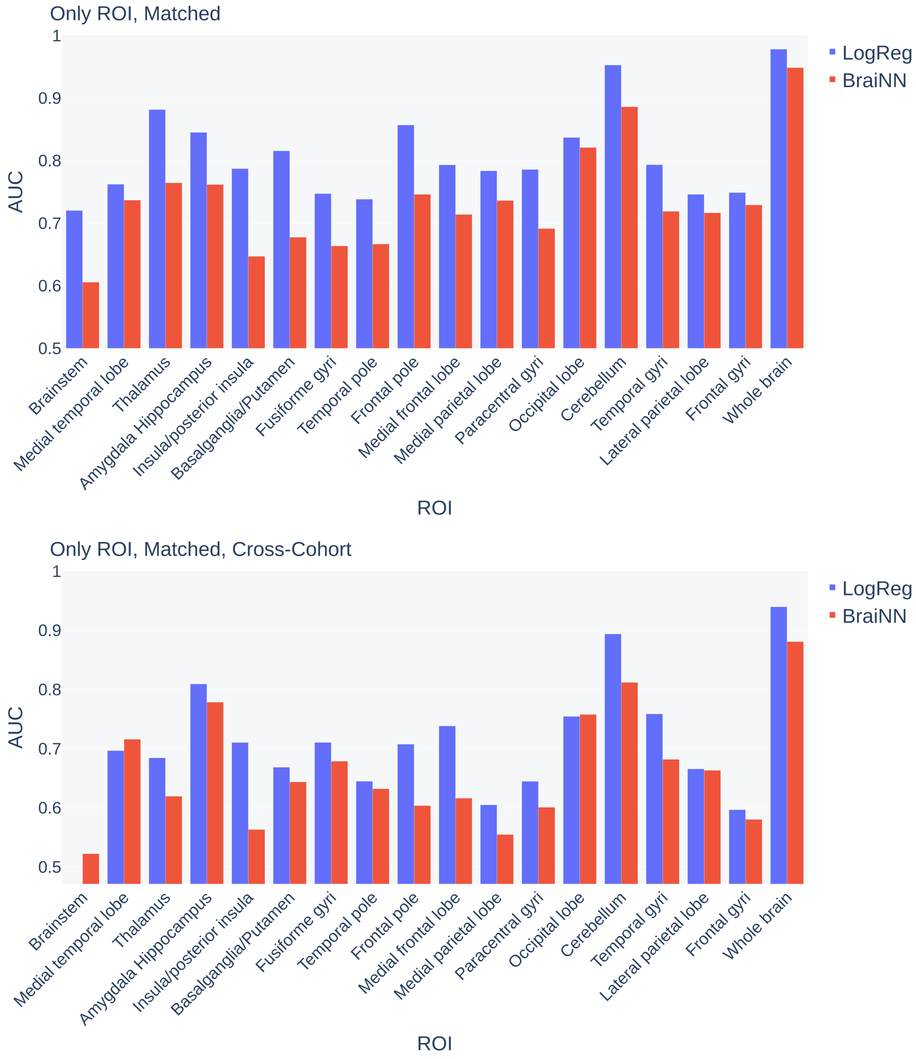

Supplementary Figure 5: AUC of LogReg and BraiNN for “whole brain” images and images containing only certain brain regions when predicting the HCP data set. Top: Performance on the test data (see Section 2.3). Bottom: Performance on the HCP data set. In all cases, the matched SHIP data set was used for training. See Table 3 for the exact values.

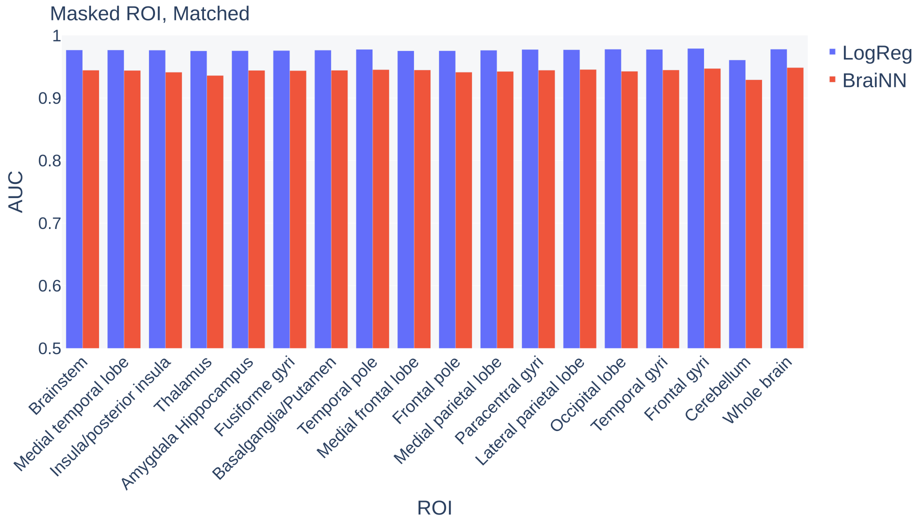

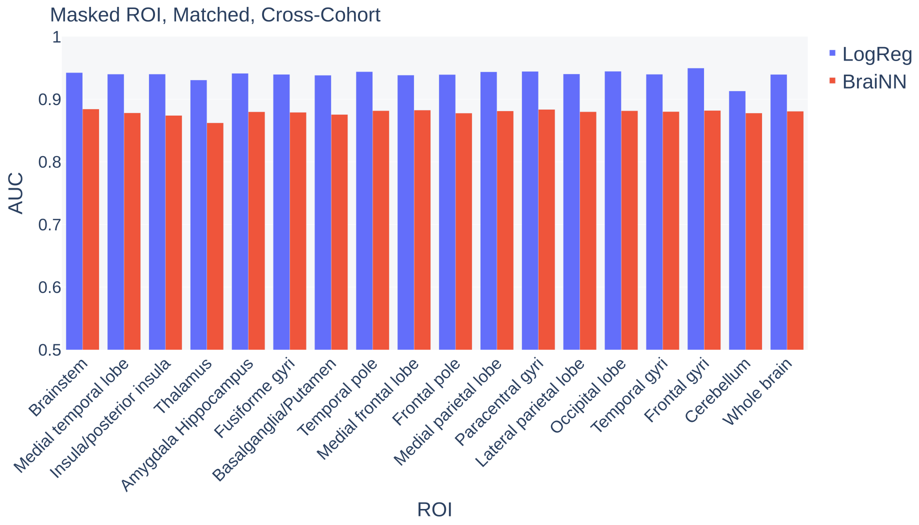

Supplementary Figure 6: AUC of LogReg and BraiNN (CNN) for "whole brain" images and images *missing* certain brain regions. Top: Performance on the test data (see Section 2.3). Bottom: Performance on the HCP data set. In all cases, the matched SHIP data set was used for training. See Table 5 for the exact values.

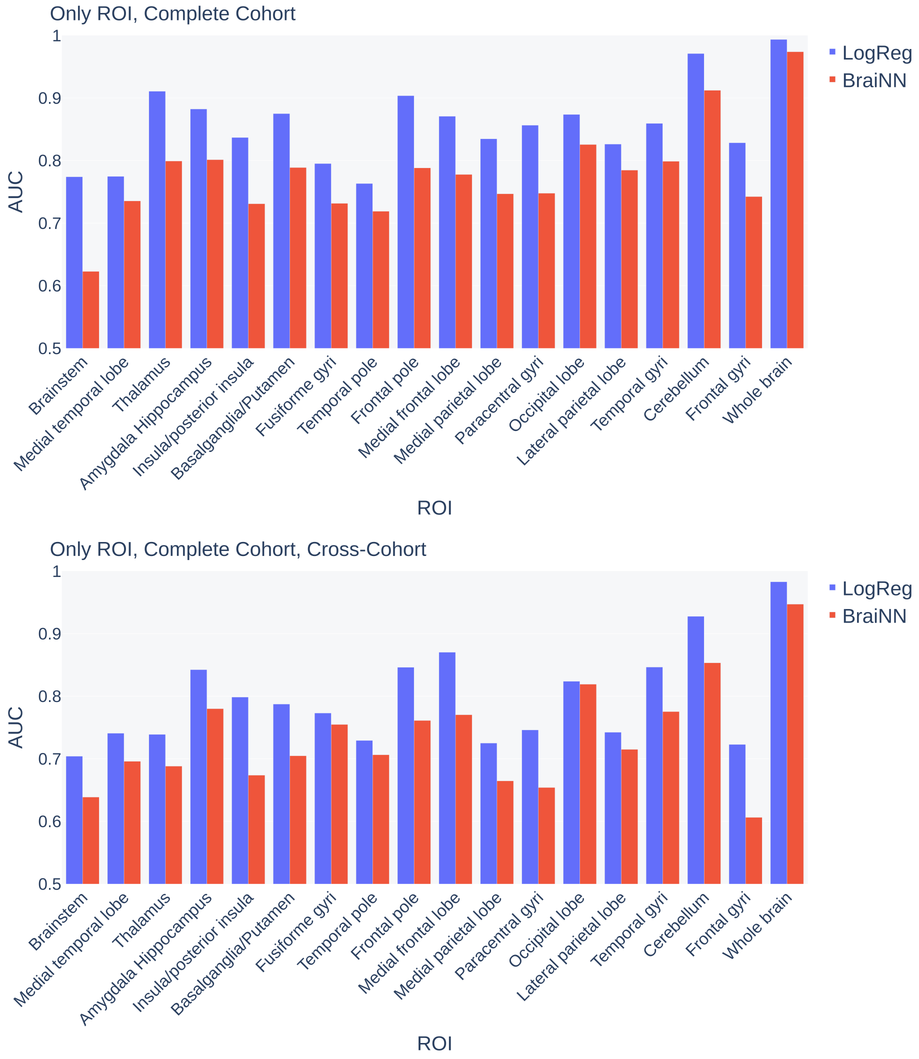

Supplementary Figure 7: AUC of LogReg and BraiNN (CNN) for "whole brain" images and images containing only certain brain regions. Top: Performance on the test data (see Section 2.3). Bottom: Performance on the HCP data set. In all cases, the complete SHIP data set was used for training. See Table 2 for the exact values.

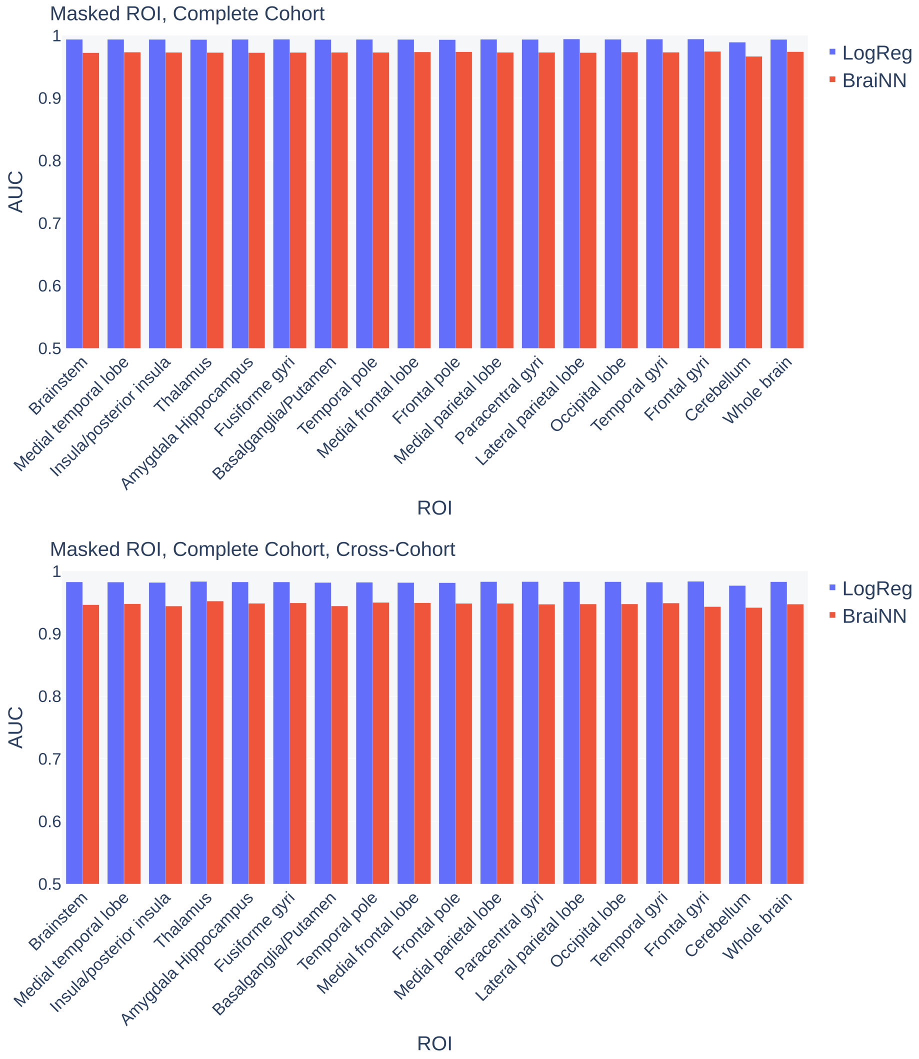

Supplementary Figure 8: AUC of LogReg and BraiNN (CNN) for "whole brain" images and images *missing* certain brain regions. Top: Performance on the test data (see Section 2.3). Bottom: Performance on the HCP data set. In all cases, the complete SHIP data set was used for training. See Table 4 for the exact values.

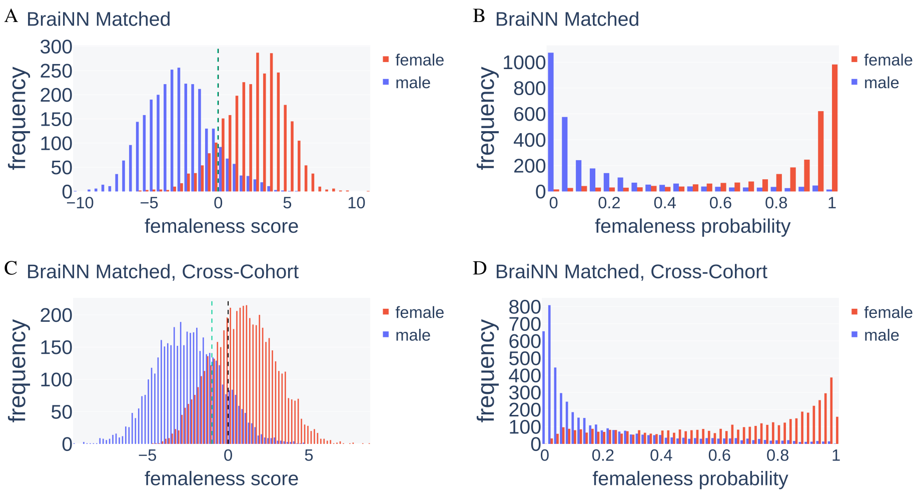

Supplementary Figure 9: Distribution of femaleness score and femaleness probability from BraiNN on SHIP test images and the HCP data set after training on the matched SHIP data set. This corresponds to Figure 5 of the main text, when BraiNN is used instead of LogReg.

Supplementary Table 1: Performance of LogReg and BraiNN in different training scenarios ('Training Data') *on differently preprocessed images.* Here, the HCP data set has been preprocessed differently from the SHIP dataset. For the HCP cohort prior to spatial normalization a cohort-specific template was calculated using the DARTEL template creation procedure of SPM. The spatially normalized GM images were modulated in a way, that each voxel encodes the local GMV prior to spatial normalization. In all cases, SHIP data was used for training. Consequently, HCP evaluations are cross-cohort. In the 'Complete SHIP' case, the entire SHIP data set was used for training. For 'Matched SHIP', the modified SHIP data set with equal TIV distributions for males and females was used. In the 'Reduced SHIP' scenario, randomly sampled subsets of the entire SHIP data set were used with the same size as the 'Matched SHIP' data set. Accuracy denotes the fraction of correctly classified MRI scans at a femaleness score threshold of 0. AUC is the area under the receiver operating characteristic curve and not sensitive to the threshold. Evaluations on SHIP were done using respectively held-back test data.

Training Data	Model	SHIP Accuracy	HCP Accuracy	SHIP AUC	HCP AUC
Complete SHIP	LogReg	95.25%	57.75%	0.991	0.955
	BraiNN	90.05%	84.24%	0.964	0.927
Matched SHIP	LogReg	91.49%	66.02%	0.971	0.845
	BraiNN	86.14%	72.19%	0.933	0.834
Reduced SHIP	LogReg	92.57%	57.28%	0.984	0.942
	BraiNN	87.57%	81.25%	0.950	0.907

Supplementary Table 2: Accuracies (acc.) and AUC for both models trained on the **complete SHIP** data set when only **certain ROIs are visible** in the images.

ROI	size in voxels	LogReg				BraiNN			
		SHIP		HCP		SHIP		HCP	
		acc.	AUC	acc.	AUC	acc.	AUC	acc.	AUC
Brainstem	504.0	0.6976	0.7740	0.6163	0.7040	0.5862	0.6227	0.5897	0.6387
Medial temporal lobe	1354.0	0.7062	0.7747	0.5793	0.7408	0.6755	0.7354	0.5587	0.6959
Thalamus	5672.0	0.8344	0.9107	0.6046	0.7389	0.7248	0.7992	0.5675	0.6882
Amygdala Hippocampus	5816.0	0.7989	0.8823	0.6633	0.8424	0.7271	0.8013	0.6040	0.7800
Insula/posterior insula	5510.0	0.7612	0.8368	0.6368	0.7985	0.6701	0.7309	0.5931	0.6737
Basalganglia/Putamen	6891.0	0.7894	0.8749	0.5966	0.7874	0.7204	0.7888	0.5656	0.7047
Fusiforme gyri	6343.0	0.7143	0.7951	0.6901	0.7730	0.6700	0.7315	0.6624	0.7548
Temporal pole	7072.0	0.6935	0.7632	0.6675	0.7292	0.6586	0.7189	0.6114	0.7063
Frontal pole	13929.0	0.8188	0.9036	0.7178	0.8462	0.7150	0.7882	0.6739	0.7612
Medial frontal lobe	13244.0	0.7864	0.8707	0.7366	0.8703	0.7075	0.7777	0.6742	0.7703
Medial parietal lobe	14112.0	0.7534	0.8347	0.6633	0.7250	0.6840	0.7468	0.6040	0.6646
Paracentral gyri	23435.0	0.7704	0.8564	0.5806	0.7460	0.6816	0.7477	0.5784	0.6541
Occipital lobe	28197.0	0.7885	0.8736	0.7038	0.8238	0.7463	0.8256	0.7261	0.8192
Lateral parietal lobe	28126.0	0.7446	0.8262	0.6700	0.7423	0.7088	0.7846	0.6557	0.7150
Temporal gyri	32958.0	0.7744	0.8593	0.7635	0.8465	0.7219	0.7988	0.7001	0.7754
Cerebellum	47928.0	0.9052	0.9710	0.7691	0.9277	0.8303	0.9122	0.7710	0.8534
Frontal gyri	44235.0	0.7438	0.8284	0.6529	0.7229	0.6740	0.7424	0.5693	0.6063
Whole brain	426812.0	0.9578	0.9935	0.9188	0.9828	0.9140	0.9739	0.8517	0.9471

Supplementary Table 3: Accuracies (acc.) and AUC for both models trained on the **volume-matched SHIP** data set when only **certain ROIs are visible** in the images.

**ROI**	**size in voxels**	**LogReg**				**BraiNN**			
		**SHIP**		**HCP**		**SHIP**		**HCP**	
		**acc.**	**AUC**	**acc.**	**AUC**	**acc.**	**AUC**	**acc.**	**AUC**
Brainstem	504.0	0.6568	0.7201	0.5360	0.4713	0.5621	0.6055	0.5140	0.5223
Medial temporal lobe	1354.0	0.6980	0.7621	0.5712	0.6965	0.6827	0.7367	0.5480	0.7158
Thalamus	5672.0	0.7983	0.8814	0.6034	0.6843	0.6877	0.7644	0.5732	0.6195
Amygdala Hippocampus	5816.0	0.7624	0.8449	0.6628	0.8092	0.6928	0.7616	0.6108	0.7784
Insula/posterior insula	5510.0	0.7146	0.7870	0.6222	0.7103	0.5963	0.6468	0.5509	0.5633
Basalganglia/Putamen	6891.0	0.7276	0.8154	0.6197	0.6685	0.6329	0.6774	0.5838	0.6437
Fusiforme gyri	6343.0	0.6751	0.7471	0.6348	0.7104	0.6226	0.6634	0.6168	0.6787
Temporal pole	7072.0	0.6786	0.7382	0.5856	0.6448	0.6103	0.6665	0.5837	0.6321
Frontal pole	13929.0	0.7764	0.8568	0.6541	0.7074	0.6806	0.7459	0.5506	0.6036
Medial frontal lobe	13244.0	0.7112	0.7930	0.6676	0.7382	0.6554	0.7138	0.6001	0.6162
Medial parietal lobe	14112.0	0.7067	0.7835	0.5834	0.6048	0.6779	0.7361	0.5467	0.5548
Paracentral gyri	23435.0	0.7057	0.7857	0.4955	0.6447	0.6408	0.6913	0.5391	0.6008
Occipital lobe	28197.0	0.7539	0.8369	0.6844	0.7544	0.7430	0.8208	0.6667	0.7577
Cerebellum	47928.0	0.8821	0.9526	0.7399	0.8937	0.8019	0.8859	0.7236	0.8118
Temporal gyri	32958.0	0.7067	0.7933	0.6848	0.7586	0.6602	0.7187	0.6329	0.6818
Lateral parietal lobe	28126.0	0.6789	0.7460	0.6121	0.6657	0.6556	0.7164	0.6016	0.6632
Frontal gyri	44235.0	0.6837	0.7488	0.5645	0.5966	0.6724	0.7290	0.5508	0.5803
Whole brain	426812.0	0.9271	0.9780	0.8465	0.9396	0.8806	0.9485	0.7637	0.8808

Supplementary Table 4: Accuracies (acc.) and AUC for both models trained on the **complete SHIP** data set when certain **ROIs are not visible** in the images.

ROI	size in voxels	LogReg				BraiNN			
		SHIP		HCP		SHIP		HCP	
		acc.	AUC	acc.	AUC	acc.	AUC	acc.	AUC
Brainstem	504.0	0.9595	0.9936	0.9180	0.9826	0.9109	0.9722	0.8425	0.9461
Medial temporal lobe	1354.0	0.9583	0.9936	0.9167	0.9823	0.9130	0.9731	0.8463	0.9476
Insula/posterior insula	5510.0	0.9596	0.9935	0.9166	0.9817	0.9122	0.9728	0.8439	0.9440
Thalamus	5672.0	0.9592	0.9933	0.8671	0.9833	0.9115	0.9726	0.8764	0.9520
Amygdala Hippocampus	5816.0	0.9595	0.9936	0.9182	0.9826	0.9117	0.9724	0.8429	0.9484
Fusiforme gyri	6343.0	0.9608	0.9938	0.9160	0.9825	0.9134	0.9727	0.8565	0.9490
Basalganglia/Putamen	6891.0	0.9582	0.9933	0.9137	0.9816	0.9138	0.9729	0.8481	0.9442
Temporal pole	7072.0	0.9603	0.9936	0.9176	0.9820	0.9144	0.9728	0.8559	0.9498
Medial frontal lobe	13244.0	0.9590	0.9935	0.9132	0.9816	0.9148	0.9735	0.8480	0.9492
Frontal pole	13929.0	0.9576	0.9931	0.9161	0.9813	0.9149	0.9737	0.8613	0.9483
Medial parietal lobe	14112.0	0.9594	0.9937	0.9187	0.9830	0.9132	0.9728	0.8509	0.9483
Paracentral gyri	23435.0	0.9600	0.9936	0.9154	0.9831	0.9155	0.9729	0.8540	0.9469
Lateral parietal lobe	28126.0	0.9610	0.9941	0.9192	0.9829	0.9120	0.9725	0.8452	0.9473
Occipital lobe	28197.0	0.9602	0.9937	0.9194	0.9829	0.9115	0.9732	0.8433	0.9474
Temporal gyri	32958.0	0.9626	0.9940	0.9174	0.9823	0.9135	0.9730	0.8574	0.9488
Frontal gyri	44235.0	0.9605	0.9941	0.9193	0.9835	0.9142	0.9744	0.8316	0.9431
Cerebellum	47928.0	0.9461	0.9891	0.9008	0.9768	0.8981	0.9665	0.8505	0.9416
Whole brain	426812.0	0.9578	0.9935	0.9188	0.9828	0.9140	0.9739	0.8517	0.9471

Supplementary Table 5: Accuracies (acc.) and AUC for both models trained on the **volume-matched SHIP** data set when certain **ROIs are not visible** in the images.

ROI	size in voxels	LogReg				BraiNN			
		SHIP		HCP		SHIP		HCP	
		acc.	AUC	acc.	AUC	acc.	AUC	acc.	AUC
Brainstem	504.0	0.9247	0.9767	0.8468	0.9424	0.8703	0.9444	0.7674	0.8843
Medial temporal lobe	1354.0	0.9266	0.9766	0.8417	0.9400	0.8722	0.9440	0.7616	0.8782
Insula/posterior insula	5510.0	0.9275	0.9764	0.8430	0.9401	0.8650	0.9411	0.7576	0.8741
Thalamus	5672.0	0.9201	0.9752	0.8369	0.9307	0.8653	0.9359	0.7734	0.8622
Amygdala Hippocampus	5816.0	0.9197	0.9755	0.8417	0.9413	0.8708	0.9440	0.7611	0.8799
Fusiforme gyri	6343.0	0.9220	0.9757	0.8462	0.9397	0.8720	0.9437	0.7541	0.8791
Basalganglia/Putamen	6891.0	0.9221	0.9764	0.8426	0.9383	0.8708	0.9442	0.7675	0.8756
Temporal pole	7072.0	0.9305	0.9776	0.8513	0.9440	0.8722	0.9452	0.7666	0.8817
Medial frontal lobe	13244.0	0.9194	0.9753	0.8351	0.9385	0.8758	0.9447	0.7653	0.8826
Frontal pole	13929.0	0.9240	0.9754	0.8525	0.9394	0.8676	0.9412	0.7591	0.8778
Medial parietal lobe	14112.0	0.9263	0.9763	0.8495	0.9437	0.8664	0.9424	0.7698	0.8812
Paracentral gyri	23435.0	0.9232	0.9775	0.8568	0.9444	0.8719	0.9444	0.7764	0.8837
Lateral parietal lobe	28126.0	0.9232	0.9771	0.8406	0.9403	0.8751	0.9455	0.7664	0.8800
Occipital lobe	28197.0	0.9287	0.9779	0.8435	0.9446	0.8696	0.9426	0.7653	0.8816
Temporal gyri	32958.0	0.9280	0.9775	0.8510	0.9399	0.8688	0.9447	0.7705	0.8802
Frontal gyri	44235.0	0.9259	0.9791	0.8431	0.9497	0.8714	0.9471	0.7550	0.8820
Cerebellum	47928.0	0.8854	0.9607	0.7822	0.9131	0.8482	0.9291	0.7569	0.8779
Whole brain	426812.0	0.9271	0.9780	0.8465	0.9396	0.8806	0.9485	0.7637	0.8808
